# Snare and balloon technique for torn coronary wire retrieval from left anterior descending artery

**DOI:** 10.1093/ehjcr/ytag256

**Published:** 2026-04-16

**Authors:** Sudipta Mondal, Nadeem Afroz Muslim

**Affiliations:** Department of Cardiology, The Mission Hospital, Immon Kalyan Sarani, Sector IIC, Bidhannagar, Durgapur, West Bengal 713212, India; Department of Cardiology, The Mission Hospital, Immon Kalyan Sarani, Sector IIC, Bidhannagar, Durgapur, West Bengal 713212, India

## Case

**Figure 1:**
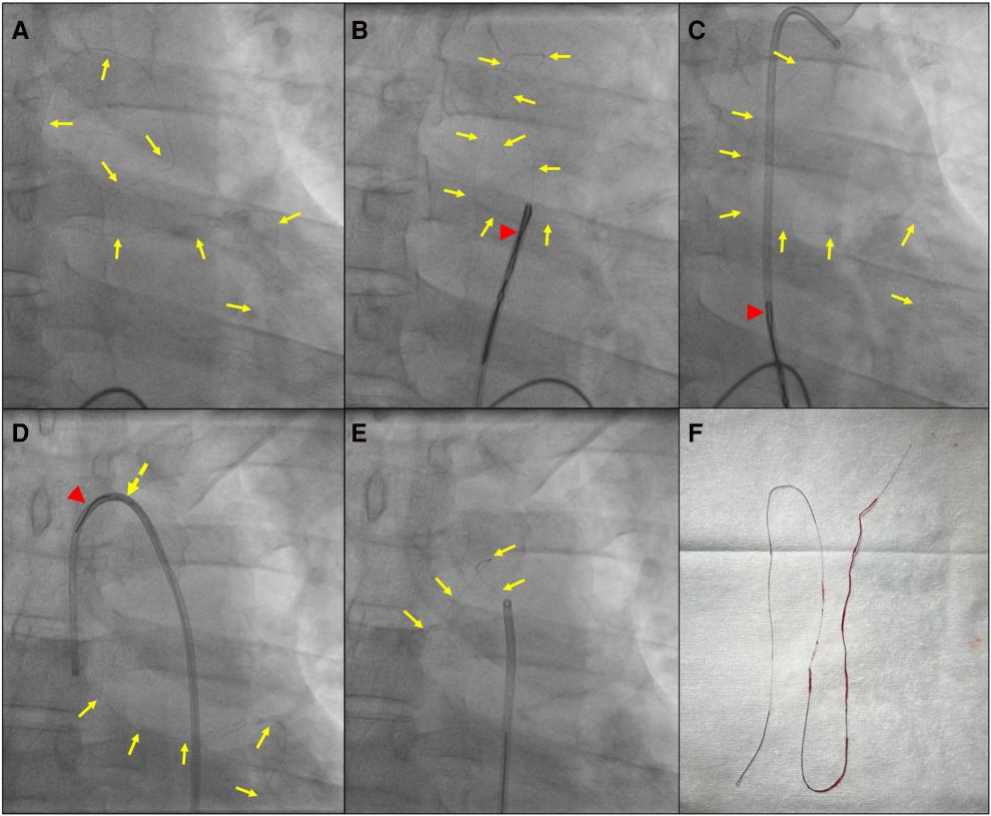
(*A*) Retention of a fractured coronary wire extending from the descending thoracic aorta to the left anterior descending artery. (*B*) The free-floating segment within the descending thoracic aorta was caught using a Medtronic Amplatzer 15 mm Goose Neck Snare. (*C*) A 6Fr multipurpose (MPA) guide catheter (Cordis) was advanced over the snared wire until it reached the ascending aorta. (*D*) A Runthrough coronary guidewire (Terumo Interventional Systems) was subsequently positioned in the distal portion of the guide catheter. (*E*) A 2 mm non-compliant balloon (NC Trek, Abbott) was then advanced over the Runthrough wire and inflated to 14 atm, effectively trapping the previously snared coronary wire between the balloon and the guide catheter. (*F*) Successful retrieval of the 20 cm coronary wire intact. In the present case, stenting the distal portion of the guidewire against the vessel wall would have secured the distal segment; however, the proximal portion of the fractured wire would have remained unconstrained and free-floating within the descending thoracic aorta. Conventional snaring was deemed insufficient, as it failed to provide the necessary traction for secure advancement into the guide catheter. Alternative manoeuvres, such as multi-wire entanglement, were considered unsuitable given the mobility of the aortic segment and the requirement for controlled, *en bloc* retrieval. Furthermore, neither deep-guide wedging nor balloon trapping could guarantee stable capture of the mobile proximal end. Although surgical intervention is documented for cases with aortic extension, it was not pursued as the primary strategy in light of the patient’s haemodynamic stability and the perceived feasibility of a percutaneous approach. Arrow—trapped torn coronary wire; arrowhead—snare; dashed arrow—balloon.

A sexagenarian patient with a history of Type 2 diabetes mellitus and chronic coronary syndrome, exhibiting normal left ventricular function, was transferred from an outlying medical facility. The transfer was necessitated by the fracture and subsequent retention of a coronary guidewire during a percutaneous coronary intervention. The retained guidewire fragment extended from the descending thoracic aorta (DTA) to the left anterior descending (LAD) artery (*Figure 1A*, arrows; Supplementary material online, *Video S1*). The free-floating segment within the DTA was caught using a Medtronic Amplatzer 15 mm Goose Neck Snare (*Figure 1B*; Supplementary material online, *Video S2*). The wire was then manoeuvred into a 6Fr multipurpose (MPA) guide catheter (Cordis).

However, repeated attempts to advance the coronary wire through the guide catheter were unsuccessful because the snare failed to provide adequate grip. Consequently, the guide catheter was advanced over the snared wire until it reached the ascending aorta (*Figure 1C*). A Runthrough coronary guidewire (Terumo Interventional Systems) was subsequently positioned in the distal portion of the guide catheter (*Figure 1D*). A 2 mm non-compliant balloon (NC Trek, Abbott) was then advanced over the Runthrough wire and inflated to 14 atm, effectively trapping the previously snared coronary wire between the balloon and the guide catheter (*Figure 1D* and *E*; Supplementary material online, *Video S3*).

With the wire firmly secured, the entire system was carefully withdrawn, resulting in the successful retrieval of the 20 cm coronary wire intact (*Figure 1E* and *F*). Subsequently, the patient underwent coronary angioplasty of the right coronary artery, and a moderate lesion was identified in the LAD artery by fractional flow reserve analysis.

The retained coronary wire presented a significant challenge due to the poor visibility of its stretched radiopaque segment. This necessitated performing the entire retrieval procedure at a high frame rate using fluoroscopy to ensure adequate visualization and precise manipulation.

A free end of the torn wire floating in the DTA precluded most retrieval techniques (stenting against the vessel wall, snare loop, multi-wire technique, ‘knuckle twister’ technique, bioptome, microcatheter trapping, deep guide catheter wedging with balloon inflation, and pigtail catheter entrapment) due to difficulties in catching the wire and removing the fragmented wire en masse. In summary, this case illustrates the practical application of a combined ‘snare and balloon technique’ for successful percutaneous retrieval of a fractured retained coronary wire from the LAD to the DTA.^1–3^

## Supplementary Material

ytag256_Supplementary_Data

## Data Availability

Data are available on request from the authors.
